# Analysis of feedback loops and robustness in network evolution based on Boolean models

**DOI:** 10.1186/1471-2105-8-430

**Published:** 2007-11-07

**Authors:** Yung-Keun Kwon, Kwang-Hyun Cho

**Affiliations:** 1 Department of Bio and Brain Engineering and KI for the BioCentury, Korea Advanced Institute of Science and Technology, 335 Gwahangno, Yuseong-gu, Daejeon, 305-701, Republic of Korea

## Abstract

**Background:**

Many biological networks such as protein-protein interaction networks, signaling networks, and metabolic networks have topological characteristics of a scale-free degree distribution. Preferential attachment has been considered as the most plausible evolutionary growth model to explain this topological property. Although various studies have been undertaken to investigate the structural characteristics of a network obtained using this growth model, its dynamical characteristics have received relatively less attention.

**Results:**

In this paper, we focus on the robustness of a network that is acquired during its evolutionary process. Through simulations using Boolean network models, we found that preferential attachment increases the number of coupled feedback loops in the course of network evolution. Whereas, if networks evolve to have more coupled feedback loops rather than following preferential attachment, the resulting networks are more robust than those obtained through preferential attachment, although both of them have similar degree distributions.

**Conclusion:**

The presented analysis demonstrates that coupled feedback loops may play an important role in network evolution to acquire robustness. The result also provides a hint as to why various biological networks have evolved to contain a number of coupled feedback loops.

## Background

There is a growing interest in understanding the principle of biological network evolution and many network growth models have been proposed to investigate this issue. For example, the duplication-mutation models suggest that network growth occurs through the duplication of an existing node and mutation of links by deleting an existing link or adding a new link [1,2]. In addition, other models such as random static network models where links are randomly connected [3,4], aging vertex network models where the probability of producing new edges decreases with the age of a network node [5], and small-world network models based on an interpolation between regular ring lattices and randomly connected graphs [6], have been introduced. Meanwhile, there have been various studies on the topological properties of biological networks, and one prominent result is about the scale-free property indicating the power-law distribution in the number of connections (degree) per network node [7]. In this regard, finding a network growth model that can produce a scale-free network has become an issue. Preferential attachment, a way of adding new interactions to a network node in proportion to the connectivity of the node (i.e. the number of links connected to the node), has been considered the most plausible growth model [8], and it has been partially supported by showing that old proteins or genes are likely to have high connectivity in many biological networks [9,13]. According to preferential attachment, the motive of evolution is only connectivity, which is therefore regarded as the most important factor characterizing the biological networks. However, this approach only focuses on the topological characteristics of networks and there have been other studies showing that the connectivity has a limitation in explaining the entire functional or dynamical behavior of biological networks. For example, it has been shown that the connectivity of a network node is not related to its essentiality in transcriptional regulatory networks [14] and a highly connected node is not directly related to the robustness of the network [15]. In addition, the connectivity of a node cannot explain the influence of a metabolite in a phenotypic state in metabolic networks [16]. In these respects, there is a pressing need to investigate other features of network evolution that can better explain the dynamical properties of biological networks. To this end, in this paper we consider a feedback loop, a circular chain of interaction, as another important factor. Feedback loops are important because they are ubiquitously found in most biological networks. Moreover, it is intriguing that feedback loops exist in the form of multiple coupled feedback loops in many biological systems such as budding yeast polarization [17], eukaryotic chemotaxis [18], and Ca^2+ ^spikes [19]. Note that a system with multiple feedback loops is more robust than one with a single feedback loop [20-22]. In this paper, we hypothesize that coupled feedback loops affect dynamical behaviors in the course of network evolution, particularly affecting the robustness of a network. Many cellular systems are known to be considerably robust to environmental changes. For instance, the chemotaxis receptor of *Escherichia coli *maintains its tumbling frequency despite significant changes in rate constants or ligand concentrations [23]. The development of the correct segment polarity patterns in *Drosophila melanogaster *embryos is robust to the changes of the initial conditions, reaction parameters, or certain gene products [24].

To verify our hypothesis on the relationship between feedback loops and the robustness of a network, we employ random Boolean network models where the directed links between nodes are randomly chosen and then consider the evolution of biological networks that are represented by a directed graph. For example, the growth of gene regulatory networks in various organisms through the duplication of transcriptional factors or target genes can be described by using directed networks [13]. Then, we define the robustness of a Boolean network model as the probability with which either an initial state mutation or an update rule mutation does not cause the network converge to a new attractor. The 'attractor' has an important meaning in biological network dynamics. In Boolean network models, a state trajectory starts from an initial state and eventually converges to either a fixed-point or a limit-cycle attractor. Hence, these attractors represent the dynamical behaviors of biological networks such as multistability, homeostasis, and oscillations [25-27]. For example, in the regulatory network of inducing phenotype variations in bacteria, some epigenetic traits are represented by multiple fixed-point attractors [28]. This multistability is a common feature of adaptive processes in bacteria. In addition, mitogen-activated protein kinase cascades in animal cells [25,26] and cell cycle regulatory circuits in *Xenopus *and *Saccharomyces cerevisiae *[27,29] are known to produce multistable attractors. However, the transcriptional network of mRNAs for Notch signaling molecules shows an oscillation with a 2-h cycle by Hes1 transcription [30] and this corresponds to a limit-cycle attractor. Such Hes1 oscillation is found in various cell types. As illustrated by these examples, attractors represent the essential dynamics of biological networks. Therefore, converging to a different attractor due to mutations in the network can be interpreted as lacking robustness. This concept has been widely used in a number of previous studies employing computational approaches [31,34].

## Results and Discussion

### Change of robustness and the number of feedback loops during network evolution

**Figure 1:**
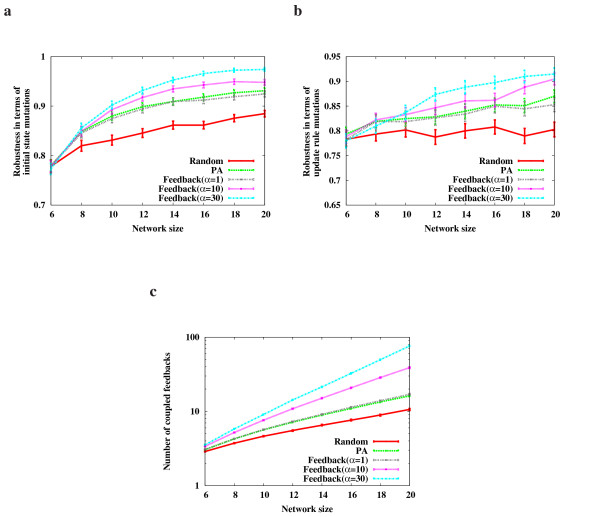
**The variation of robustness and the number of coupled feedback loops along with network evolution**. **(a) **Change of robustness with respect to initial update mutations during network evolution for the "CONJ-DISJ" model (see Methods). **(b) **Change of robustness with respect to update rule mutations during network evolution for the "CONJ-DISJ" model. **(c) **Change of the number of coupled feedback loops during network evolution for the "CONJ-DISJ" model. Here, "PA" refers to the networks that are evolved using the preferential attachment model and "Feedback" refers to the networks that are evolved by favoring the formation of coupled feedback loops with a selection pressure (*α*) (see Methods for details). In addition, "Random" denotes the randomly generated networks with the same number of nodes and links as those in "PA" and "Feedback". Network size means the number of nodes (|*V*|) of a network. For each model, the average and the confidence level (95%) of robustness over 1,000 networks are shown on the y-axis. The Boolean networks using the "CONJ" and "DISJ" models also showed similar results (see additional data file 1).

To test our hypothesis, we have performed extensive computer simulations using Boolean network models (see Methods). We have examined robustness with respect to the initial state mutations (Fig. 1a), update rule mutations (Fig. 1b), and the number of coupled feedback loops (Fig. 1c) of a network during its evolution. The simulations showed that the networks evolved by preferential attachment (PA) are more robust and produce more coupled feedback loops than random networks. This suggests that the number of coupled feedback loops might be highly correlated to the robustness of a network during evolution. To further investigate this relationship, we have examined the networks evolved by the "Feedback" model which favors a larger number of feedback loops under a selection pressure (*α*). (In this simulation, three values, 1, 10, and 30, were chosen for *α*) Compared with the networks evolved using the "PA" model, the networks evolved using the Feedback" model showed very similar results for *α *= 1 with respect to both robustness and the number of coupled feedback loops. Furthermore, the higher the selection pressure was, the larger the number of feedback loops the evolved networks had, which results in enhanced robustness. This implies that coupled feedback loops are critical in enhancing the robustness of networks during evolution. Feedback loops help maintain the stability of a network and multiple coupled feedback loops strengthen this function. We verified that this result holds irrespective of the update function used for evolution (see additional data file 1).

### Correlation between robustness and the number of feedback loops

**Figure 2:**
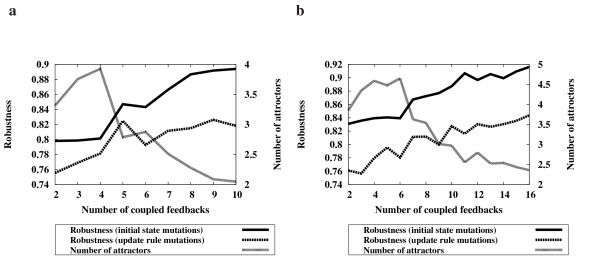
**The correlation between the number of coupled feedback loops and the robustness of the network**. **(a) **Average results from 9,000 networks with 10 nodes and 14 links. **(b) **Average results from 15,000 networks with 14 nodes and 20 links. The robustness of all the networks was computed based on the "CONJ-DISJ" model. The Boolean networks using the "CONJ" and "DISJ" models also showed similar results (see additional data file 2).

To further investigate the relationship between the number of coupled feedback loops and network robustness, we performed additional simulations. We randomly generated 9,000 Boolean networks with |*V*| = 10 and |*A*| = 14, and 15,000 Boolean networks with |*V*| = 14 and |*A*| = 20. Then, we investigated the robustness against the number of coupled feedback loops in these networks (Fig. 2, Additional file 2). We found that there is a strong positive correlation between the number of coupled feedback loops and the robustness of a network. However, we observed that the number of attractors decreases as the number of coupled feedback loops increases. In other words, networks with more coupled feedback loops have a smaller number of attractors and thereby become more robust. This result explains the reason why the "Feedback" model produced more robust networks in Fig. 1. It also explains why the networks evolved using "PA" are more robust than the random networks, since if a network is evolved using preferential attachment, then it has more coupled feedback loops than a random network. Preferential attachment adds a new link to a highly connected node with a higher probability, so it is more likely to produce new feedback loops as the highly connected nodes could already pertain to other coupled feedback loops.

### Degree distributions

**Figure 3:**
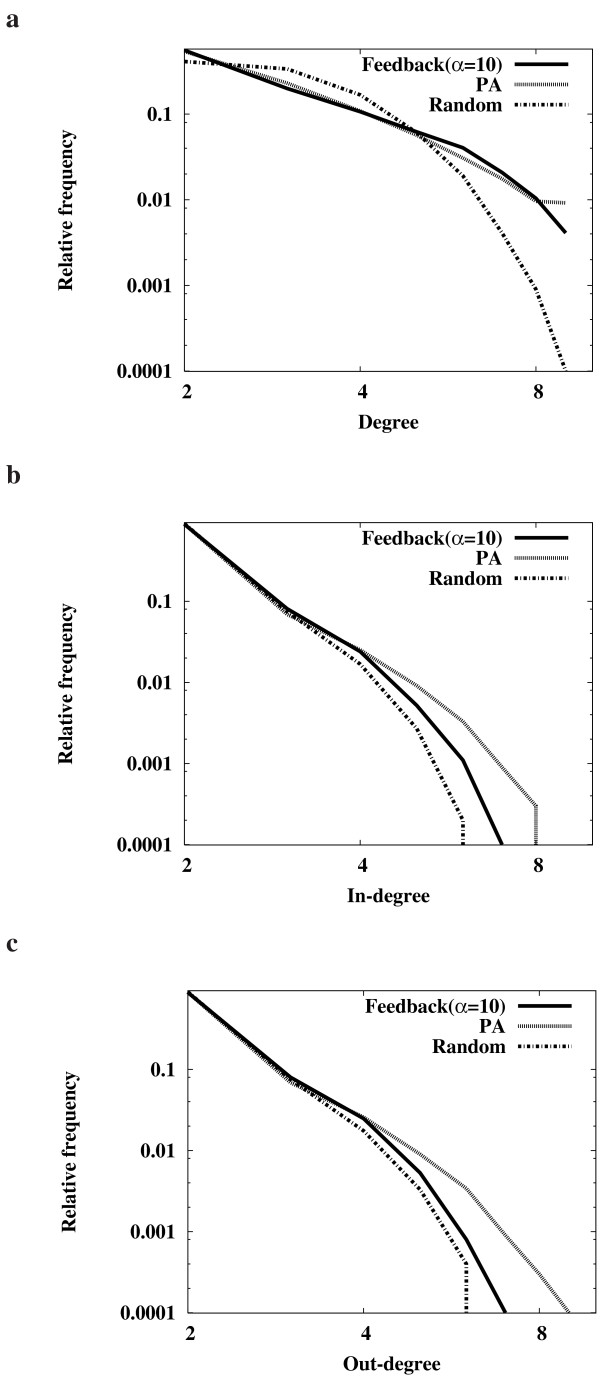
**Degree distributions of the evolved networks**. **(a) **Total degree distributions. **(b) **In-degree distributions. **(c) **Out-degree distributions. All distributions were examined over 1,000 different networks that were evolved using the "PA" and "Feedback"(*α *= 10) models until |*V*| = 46. In addition, the "Random" networks were examined. The networks evolved by the "Feedback" model with other selection pressure values also showed similar degree distributions (see additional data file 3).

In the foregoing developments, we found different dynamics between the "PA" and "Feedback" models which cannot be fully explained in terms of connectivity. We checked the degree distributions of networks obtained by "PA", "Feedback (*α *= 10)", and "Random" (Fig. 3a). The proportion of nodes with a high degree in both "PA" and "Feedback" was relatively larger than that of "Random". However, the degree distribution of "Feedback" was similar to that of "PA". We observed that the scale-free property was also preserved in the networks evolved using "Feedback". In other words, network evolution following the "Feedback" model generates a few but substantial number of highly connected hubs together with most of the other nodes only having a few links. Hence, the different characteristics of robustness between the "PA" and "Feedback" models cannot be properly explained by degree distribution alone. Furthermore, we checked the in-degree and out-degree distributions separately (Fig. 3b and Fig. 3c, respectively, Additional file 3). Compared with the total degree distribution, we found a significant difference between "Feedback" and "PA". The proportion of nodes with a high in-degree or out-degree in "Feedback" was smaller than that of "PA". This implies that the difference between the in-degree and out-degree of the hub nodes in the networks evolved using "Feedback" is smaller than that in the networks evolved using "PA". This may occur because "Feedback" attaches new links to a node in the direction towards forming a feedback loop and this results in balance between the in- and out-degrees, while "PA" introduces new links in a random direction.

### Coupled feedback loops in the evolution of biological networks

The simulation results have shown that the number of coupled feedback loops is positively correlated with the robustness of networks and, therefore, it might guide the direction of the network evolution. The strong relationship between coupled feedback loops and the robust behavior of a network is partially supported by previous experiments on various biological networks. For example, it was found that three distinct feedback loops responsible for genetic regulation, mRNA attenuation, and enzyme inhibition that regulate tryptophan concentrations in *Escherichia coli*. The complex regulatory network formed by the feedback loops induces a rapid and stable response, while being robust against uncertainties [22]. Such coupled feedback loop-based mechanisms were also observed in many other regulatory networks including the regulation of arabinose uptake in *Escherichia coli *[35], the regulation of galactose uptake [36] and the osmotic effect [37] in *Saccharomyces cerevisiae*, and the regulation of insulin signaling pathways [38]. Another example is the circadian clock consisting of two interlinked transcriptional feedback loops [39,40]. These multiple coupled feedback loops enhance the robustness of the oscillators in producing accurate circadian rhythms. In the *Drosophila *segment polarity network, it was shown that three feedback loops are necessary and sufficient to ensure the robustness of pattern formation [41]. The bacterial chemotaxis pathways in *Escherichia coli *and *Bacillus subtilis *were compared [42] and it was revealed that the core control strategy for signal processing is realized by the same feedback loop in both organisms. Moreover, in *Bacillus subtilis*, there are two additional feedback loops and these provide an additional layer of robustness that might have been acquired through evolution. Recent studies on network fragility also provide a further insight into the important roles of feedback loops in biological networks. In an uncontrolled tumor growth, feedback introduces fragilities such as the possibility of self-sustaining and cascading failures [43]. A high gain in negative feedback loops leads to steady-state stability, but fragilities might cause potentially inaccurate transient responses since the time-varying perturbations can be amplified [44]. These examples and our own simulation results lead us to infer importance of the role of multiple feedback loops in robust biological dynamics.

**Figure 4:**
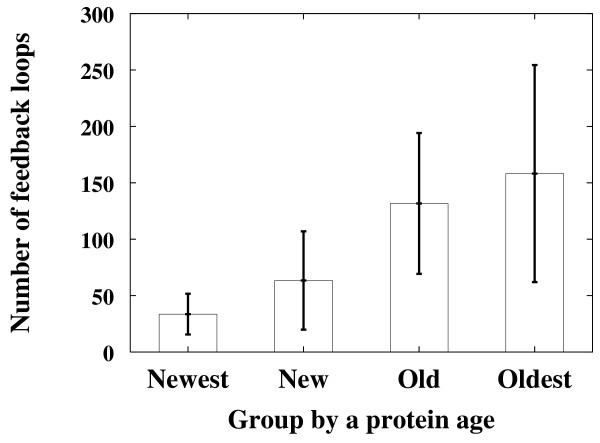
**The correlation between the number of feedback loops and the protein age of the neuronal signaling network**. The number of feedback loops are plotted against the age of the proteins. The proteins are grouped into four categories according to their estimated ages: "Newest", "New", "Old", and "Oldest" (see Methods for details). For each protein group, the average and the confidence level (95%) of the NuFBL are shown on the y-axis.

As previously mentioned, if a biological network has been evolved to contain many coupled feedback loops for robustness, the old nodes in the network are involved with a relatively larger number of feedback loops compared with the new nodes. For verification, we have examined the signaling network of the hippocampal CA1 neuron of mice [45]. The number of coupled feedback loops involved with the proteins was plotted against the estimated age of the grouped proteins as described in Methods. It was found that older proteins tend to have a larger number of coupled feedback loops (Fig. 4).

## Conclusion

In this paper, we have discovered that coupled feedback loops play an important role in enhancing the robustness of a network during its evolution. Preferential attachment, which has been known to generate scale-free properties, brings about coupled feedback loops and thereby also results in robust networks. However, if networks are evolved using a method that is biased towards having coupled feedback loops, this results in much more robust networks than those evolved using preferential attachment, while preserving almost the same degree distribution. Our study suggests that coupled feedback loops might be a critical factor in determining robust dynamics during network evolution.

Unfortunately, there are few large-scale biological networks with information about feedback loops at present. To obtain a deeper insight into network evolution with regard to the formation of feedback loops, we need to further investigate real large-scale biological networks. In addition, we need to note that there are some biological networks which do not follow preferential attachment models such as protein-protein interaction networks where proteins can have simultaneous interactions with other proteins. Therefore, future study should include the development of new network evolution models reflecting both the structural and the dynamical characteristics of biological networks and the analysis of real large-scale biological networks.

## Methods

### Boolean network models

A Boolean network is represented by a directed graph *G *= (*V, A*) where *V *is a set of Boolean variables and *A *is the set of ordered pairs of the variables called directed links. Each *v*_*i *_∈ *V *has a value of 1 ("on") or 0 ("off") which represents the possible states of the corresponding elements, e.g. in gene networks, the value 1 represents the 'turn-on' status in which a gene is expressed. A directed link (*v*_*i*_, *v*_*j*_) has a positive ("activating") or negative ("inhibiting") relationship from *v*_*i *_to *v*_*j*_. The value of each variable *v*_*i *_at time *t *+ 1 is determined by the values of *k*_*i *_other variables $v_{i_{1}},v_{i_{2}},\cdots ,v_{i_{k_{i}}}$ with a link to *v*_*i *_at time *t *by the Boolean function $f_{i}:{\left\{0,1\right\}}^{k_{i}}\to \{0,1\}$. Hence, we can write the update rule as *v*_*i*_(*t *+ 1) = $f_{i}(v_{i_{1}}(t),v_{i_{2}}(t),\cdots ,v_{i_{k_{i}}}(t))$ where we use either a logical conjunction or disjunction for all signed relationships in *f*_*i*_. For example, if a Boolean variable *v *has a positive relationship from *v*_1_, a negative relationship from *v*_2_, and a positive relationship from *v*_3_, the conjunction and disjunction update rules are *v*(*t *+ 1) = *v*_1_(*t*) ∧ $\bar{v_{2}}$(*t*) ∧ *v*_3_(*t*) and *v*(*t *+ 1) = *v*_1_(*t*) ∨ $\bar{v_{2}}$(*t*) ∨ *v*_3_(*t*), respectively. Then, in the case of a conjunction, the value of *v *at time *t *+ 1 is 1 only if the values of *v*_1_, *v*_2_, and *v*_3 _at time *t *are 1, 0, and 1, respectively. Whereas in the case of a disjunction, the value of *v *at time *t *+ 1 is 1 if at least one of the states of the clauses, *v*_1_(*t*), $\bar{v_{2}}$(*t*), and *v*_3_(*t*) is 1. In many previous studies, biological networks were successfully described by Boolean models using conjunction or disjunction update functions [46-49]. In this paper, we assume three models: "CONJ", "DISJ", and "CONJ-DISJ". The "CONJ" and "DISJ" models mean that every node in a network has a conjunction or disjunction update function, respectively. However, the conjunction or disjunction update function is randomly selected (with a uniform probability distribution) at each network node in the "CONJ-DISJ" model. All variables are synchronously updated.

In this paper, we only consider a connected Boolean network since a Boolean network composed of disconnected multiple subnetworks can be considered as a composition of such connected subnetworks. Given a Boolean network with *N *Boolean variables, *v*_1_, *v*_2_,⋯, *v*_*N*_, we define a *state *as a vector consisting of values of the Boolean variables: there are 2^*N *^states in total. Each state transitions to another state through a set of *N *Boolean update functions, *f*_1_, *f*_2_,⋯,*f*_*N*_. We can construct a *state transition network *that represents the transition of each state. A state trajectory starts from a state and converges to either a fixed-point or a limit-cycle attractor. These attractors can describe the various behaviors of biological systems such as multi-stability, homeostasis, and oscillations. A network is considered robust if the trajectories starting from different initial states converge to the same attractor. More specifically, we define the robustness of a network in two ways. The first definition of robustness is with respect to the initial state mutations. For this, we construct *S *with a set of pairs of states (*s*, *s'*) where a Hamming distance of *s *and *s' *is one. (The Hamming distance between two states is defined as the number of Boolean variables having different values.) Hence, there are *N*2^*N*-1 ^such pairs of states. The robustness of a network is defined as the ratio of the number of pairs of states whose trajectories converge to the same attractor to the total number of pairs of states in *S*. The initial state mutation corresponds to the abnormal state (or malfunctioning) of a protein or gene caused by mutations. The second definition is with respect to the update rule mutations. This is defined as the probability with which two state trajectories starting from the same state do not converge to different attractors where one of the two trajectories is obtained by the update rule mutation with a probability of 0.2 for the erroneous updating of *v*. The update rule mutation corresponds to the change of relationships between nodes by removing or adding links. Although the Boolean network is a highly simplified model of a real biological network, it can still capture many essential aspects of real dynamics [31]. Most of all, it allows us to investigate the dynamics of large-scale networks [46].

### Definition of feedback loops

Given a network composed of a set of nodes and a set of links between the nodes, a feedback loop is a closed simple cycle where the nodes are not revisited except the starting and ending nodes. For example, *v*_0 _→ *v*_1 _→ *v*_2 _→ ⋯ → *v*_*L*-1 _→ *v*_*L *_is a feedback loop of length *L*(≥ 1) if there are links from *v*_*i*-1 _to *v*_*i *_(*i *= 1, 2,...,*L*) with *v*_0 _= *v*_*N *_and *v*_*j *_≠ *v*_*k *_for *j*, *k *∈ {0, 1,...,*L *- 1}. The number of feedback loops in a network denotes the total number of different feedback loops.

### Evolution of Boolean networks

In our simulations, a network is evolved as follows: a small network with |*V*| = 4 and |*A*| = 5 is randomly generated. Two new nodes, *v*_*a *_and *v*_*b*_, and three links are added to the network where one link is a connection from *v*_*a *_to *v*_*b*_, another is a connection from an existing node to *v*_*a*_, and the other is a connection from *v*_*b *_to an existing node (see additional file 4 for an illustration). This forms a network with |*V*| = 6 and |*A*| = 8. By repeating this process, the network complexity can be controlled. In other words, the average number of links per node converges to approximately $\frac{3}{2}$ since |*A*| = $\frac{3}{2}$|*V*| - 1 holds.

As a network with |*V*| grows into a new network with |*V*| + 2 in the above process, *N*^2 ^number of candidate pairs of links emerge and, therefore, a selection mechanism should be involved. In this paper, two models are considered: "PA" (the traditional preferential attachment model) and "Feedback" (the newly introduced model favoring the formation of coupled feedback loops). The "PA" model represents the traditional preferential attachment mechanism and employs the fitness of a candidate network as follows:

*fitness *= *k*_*a *_+ *k*_*b*_,

where *k*_*a *_and *k*_*b *_are the connectivities of the nodes that are newly linked to *v*_*a *_and *v*_*b*_, respectively. The "Feedback" model chooses a pair of new links according to the following fitness:

*fitness *= *n*^*α*^,

where *n *is the number feedback loops of the candidate network and *α *is the selection pressure.

### Analysis of the hippocampal CA1 neuronal signaling network

We considered all 545 proteins and their 1,258 interactions in the signaling network of the hippocampal CA1 neuron of mice [45]. In Fig. 4, proteins were grouped according to their estimated ages. To estimate the protein age, we searched the orthologs of the proteins in five completely sequenced eukaryotic genomes, *Mus musculus*, *Drosophila melanogaster*, *Caenorhabditis elegans*, *Saccharomyces cerevisiae*, and *Schizosaccharomyces pombe *using the Inparanoid database [50]. We defined four protein groups as follows: the proteins present in all eukaryotes ("Oldest"); the proteins present in *Mus musculus*, *Drosophila melanogaster*, and *Caenorhabditis elegans *but absent from *Saccharomyces cerevisiae *and *Schizosaccharomyces pombe *("Old"); the proteins present in *Mus musculus *and *Drosophila melanogaster *but absent from *Caenorhabditis elegans*, *Saccharomyces cerevisiae*, and *Schizosaccharomyces pombe *("New"); the proteins present in *Mus musculus *but absent from the other four genomes ("Newest"). As it is difficult to enumerate all possible feedback loops in such a large network, we only considered the feedback loops whose length (i.e. the number of links comprising the feedback loop) is less than or equal to 10.

## Authors' contributions

YKK conceived of the study, wrote the program code, and drafted the manuscript. KHC guided the study, coordinated the project, and revised the manuscript. Both authors read and approved the final manuscript.

## Supplementary Material

Additional file 1 **The variation of robustness and the number of coupled feedback loops along with network evolution**. **(a) **Change of robustness with respect to initial state mutations with network evolution using the "CONJ" model. **(b) **Change of robustness with respect to initial state mutations with network evolution using the "DISJ" model. **(c) **Change of robustness with respect to update rule mutations with network evolution using the "CONJ" model. **(d) **Change of robustness with respect to update rule mutations with network evolution using the "DISJ" model. **(e) **Change of the number of coupled feedback loops with network evolution using the "CONJ" model. **(f) **Change of the number of coupled feedback loops with network evolution using the "DISJ" model. All results were averaged over 1,000 networks. Click here for file

Additional file 2 **The correlation between the number of coupled feedback loops and the robustness of a network**. **(a) **Average results from 9,000 networks with 10 nodes and 14 links using the "CONJ" model. **(b) **Average results from 16,000 networks with 14 nodes and 20 links using the "CONJ" model. **(c) **Average results from 9,000 networks with 10 nodes and 14 links using the "DISJ" model **(d) **Average results from 16,000 networks with 14 nodes and 20 links using the "DISJ" model. Click here for file

Additional file 3 **Degree distributions of the evolved networks**. **(a) **Total degree distribution. **(b) **In-degree distribution. **(c) **Out-degree distribution. All distributions were examined over 1,000 different networks that were evolved using "Feedback" models until |*V*| = 46. Click here for file

Additional file 4 **Illustration of the network evolution process**. Given a network with four nodes, *v*_1_, *v*_2_, *v*_3_, and *v*_4_, it grows by repetitively adding two nodes (*v*_*a *_and *v*_*b*_) and three interaction links where one link is from an existing node to a new node (i.e. from *v*_2 _to *v*_*a*_), another link is from a new node to an existing node (i.e. from *v*_*b *_to *v*_4_), and the other link is between the new nodes (i.e. from *v*_*a *_to *v*_*b*_). Click here for file
