# Supplementary figures and images for: Analysis of feedback loops and robustness in network evolution based on Boolean models
Source: BMC Bioinformatics. 2007 Nov 7;8:430. doi: 10.1186/1471-2105-8-430 (PMC2249609; doi:10.1186/1471-2105-8-430)

## Additional Data File 1

**a**

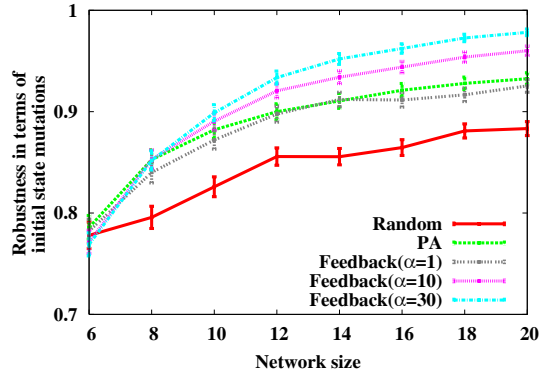

**b**

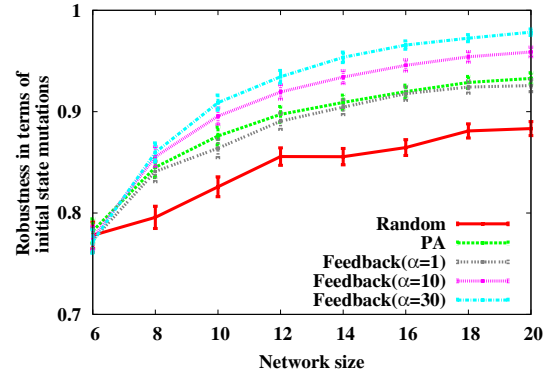

**c**

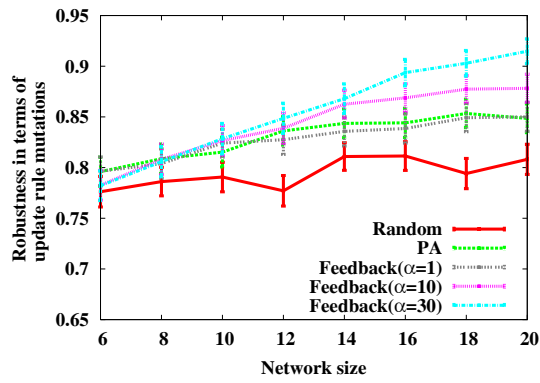

**d**

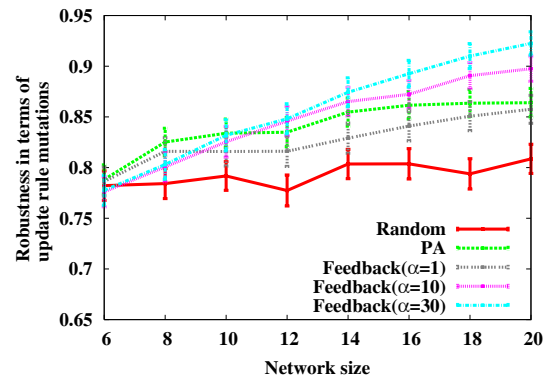

**e**

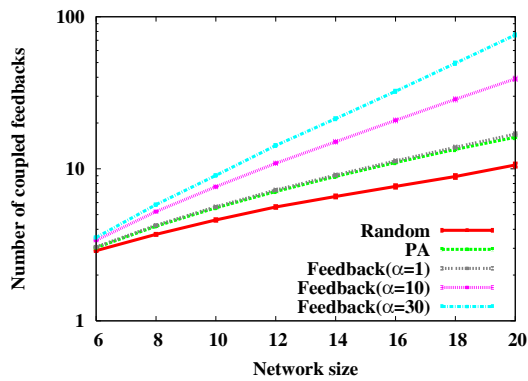

**f**

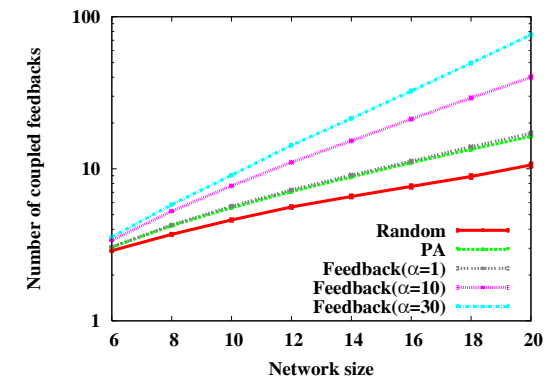

Supplement: Additional file 1 — The variation of robustness and the number of coupled feedback loops along with network evolution. (a) Change of robustness with respect to initial state mutations with network evolution using the "CONJ" model. (b) Change of robustness with respect to initial state mutations with network evolution using the "DISJ" model. (c) Change of robustness with respect to update rule mutations with network evolution using the "CONJ" model. (d) Change of robustness with respect to update rule mutations with network evolution using the "DISJ" model. (e) Change of the number of coupled feedback loops with network evolution using the "CONJ" model. (f) Change of the number of coupled feedback loops with network evolution using the "DISJ" model. All results were averaged over 1,000 networks. [file 1471-2105-8-430-S1.pdf]

## Additional Data File 2

**a**

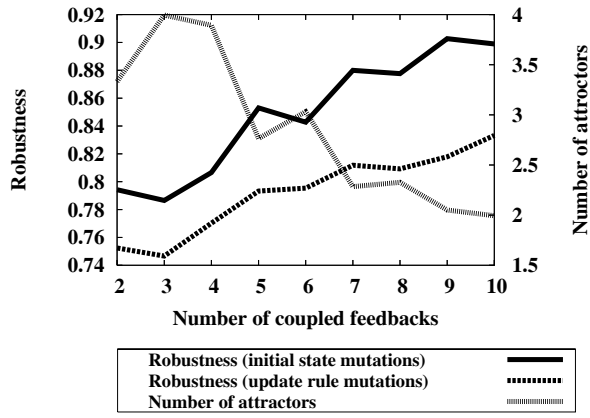

**b**

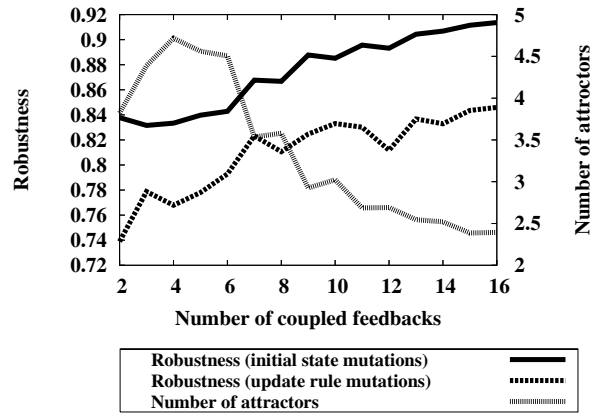

**c**

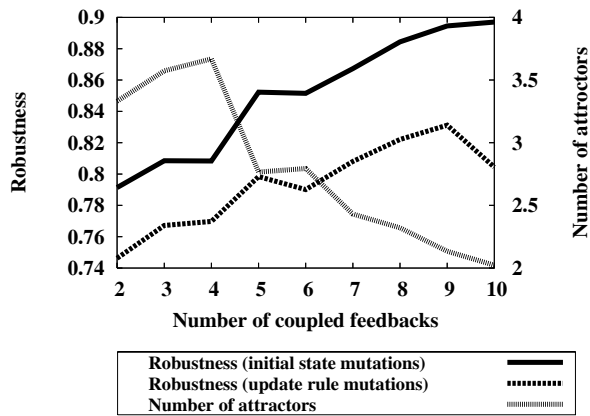

**d**

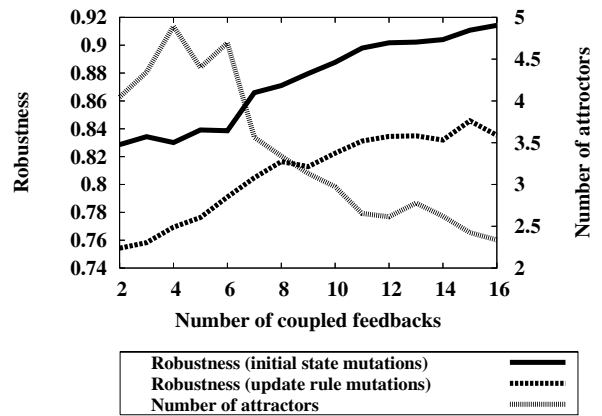

Supplement: Additional file 2 — The correlation between the number of coupled feedback loops and the robustness of a network. (a) Average results from 9,000 networks with 10 nodes and 14 links using the "CONJ" model. (b) Average results from 16,000 networks with 14 nodes and 20 links using the "CONJ" model. (c) Average results from 9,000 networks with 10 nodes and 14 links using the "DISJ" model (d) Average results from 16,000 networks with 14 nodes and 20 links using the "DISJ" model. [file 1471-2105-8-430-S2.pdf]

### Additional Data File 3

**a**

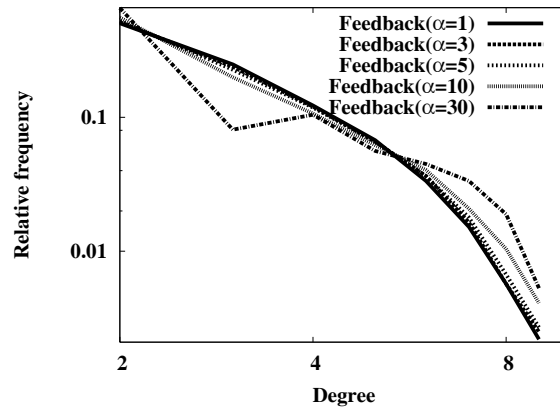

**b**

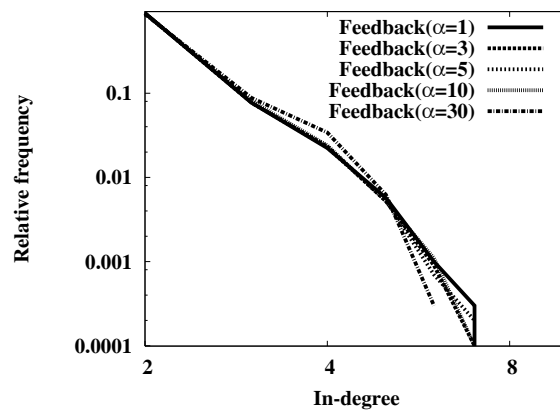

**c**

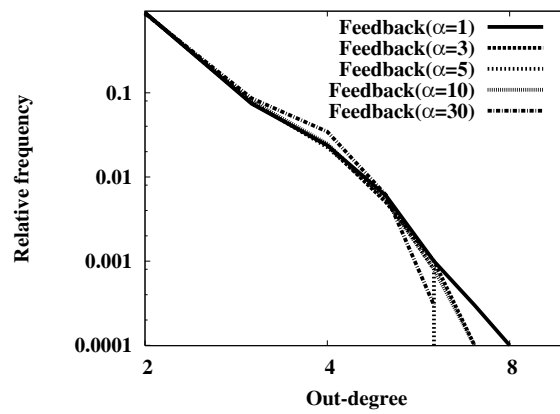

Supplement: Additional file 3 — Degree distributions of the evolved networks. (a) Total degree distribution. (b) In-degree distribution. (c) Out-degree distribution. All distributions were examined over 1,000 different networks that were evolved using "Feedback" models until |V| = 46. [file 1471-2105-8-430-S3.pdf]

# Additional Data File 4

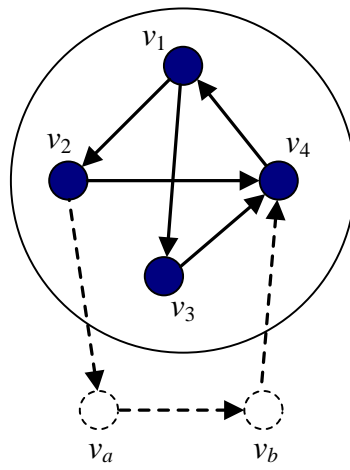

Supplement: Additional file 4 — Illustration of the network evolution process. Given a network with four nodes, v1, v2, v3, and v4, it grows by repetitively adding two nodes (va and vb) and three interaction links where one link is from an existing node to a new node (i.e. from v2 to va), another link is from a new node to an existing node (i.e. from vb to v4), and the other link is between the new nodes (i.e. from va to vb). [file 1471-2105-8-430-S4.pdf]
